# Association of body mass index with risk of acute myocardial infarction and mortality in Norwegian male and female patients with suspected stable angina pectoris: a prospective cohort study

**DOI:** 10.1186/1471-2261-14-68

**Published:** 2014-05-21

**Authors:** Heidi Borgeraas, Jens Kristoffer Hertel, Gard Frodahl Tveitevåg Svingen, Reinhard Seifert, Eva Kristine Ringdal Pedersen, Hall Schartum-Hansen, Jøran Hjelmesæth, Ottar Nygård

**Affiliations:** 1 Morbid Obesity Center, Vestfold Hospital Trust, Tønsberg, Norway; 2 Institute of Clinical Medicine, University of Oslo, Oslo, Norway; 3 Department of Clinical Science, University of Bergen, Bergen, Norway; 4 Department of Heart Disease, Haukeland University Hospital, Bergen, Norway

**Keywords:** Acute myocardial infarction, Body mass index, Cardiovascular disease, Obesity paradox

## Abstract

**Background:**

A number of previous studies have suggested that overweight or obese patients with coronary artery disease (CAD) may have lower morbidity and mortality than their leaner counterparts. Few studies have addressed possible gender differences, and the results are conflicting. We examined the association between body mass index (BMI) and risk of acute myocardial infarction (AMI), cardiovascular (CV) death and all-cause mortality in men and women with suspected stable angina pectoris.

**Method:**

The cohort included 4164 patients with suspected stable angina undergoing elective coronary angiography between 2000 and 2004. Events were registered until the end of 2006. Hazard ratios (HR) (95% confidence intervals) were estimated using Cox regression by comparing normal weight (18.5-24.9 kg/m^2^) with overweight (25–29.9 kg/m^2^) and obese (≥30 kg/m^2^) patients. Underweight (<18.5 kg/m^2^) patients were excluded from the study.

**Results:**

Of 4131 patients with complete data, 72% were males and 75% were diagnosed with significant CAD. The mean (standard deviation (SD)) age in the total population was 62 (10) years. Mean (SD) BMI was 26.8 (3.9) kg/m^2^, 34% was normal weight, 48% overweight and 19% obese. During follow up, a total of 337 (8.2%) experienced an AMI and 302 (7.3%) patients died, of whom 165 (4.0%) died from cardiovascular causes. We observed a significant interaction between BMI groups and gender with regards to risk of AMI (p = 0.011) and CV death (p = 0.031), but not to risk of all-cause mortality; obese men had a multivariate adjusted increased risk of AMI (HR 1.80 (1.28, 2.52)) and CV death (HR 1.60 (1.00, 2.55)) compared to normal weight men. By contrast, overweight women had a decreased risk of AMI (HR 0.56 (0.33, 0.98)) compared to normal weight women. The risk of all-cause mortality did not differ between BMI categories.

**Conclusion:**

Compared with normal weight subjects, obese men had an increased risk of AMI and CV death, while overweight women had a decreased risk of AMI. These findings may potentially explain some of the result variation in previous studies reporting on the obesity paradox.

**Trial registration:**

Clinicaltrials.gov Identifier: NCT00354081

## Background

Cardiovascular disease (CVD) is the leading cause of death globally: the majority dying from ischemic heart disease [1]. Overweight and obesity, most commonly defined according to body mass index (BMI), has been characterized as a major modifiable risk factor for cardiovascular (CV) morbidity and mortality by the American Heart Association and the American College of Cardiology [2].

As recently reviewed, some studies of patients with coronary artery disease (CAD) suggest that being overweight or obese has beneficial effects in terms of reduced risk of CV events and/or mortality; a phenomenon known as the obesity paradox. However, there is no broad consensus regarding the obesity paradox, as several studies are unsupportive of this conclusion [3].

Moreover, despite the fact that both body fat percentage and distribution vary by gender [4], only a limited number of studies among patients with CAD or suspected CAD have examined the association between BMI and risk of coronary events and mortality in men and women separately, and the reported results are conflicting [5-9]. There is, however, a tendency towards a non-disadvantageous [5,6,9] or even a beneficial [7] effect of overweight and obesity among women, while obesity appears to increase the risk of coronary events in men [5,6].

In the present study we examined the association between BMI and risk of incident acute myocardial infarction (AMI), CV death and all-cause mortality in a large population of men and women with suspected CAD. We hypothesized that overweight and/or obesity, as compared to normal weight, was associated with an increased risk of AMI, CV death and all-cause mortality among men, but not among women.

## Methods

### Study design and patient population

The patients recruited for the present investigation are described in detail elsewhere [10]. In brief, 4164 patients undergoing elective coronary angiography for suspected stable angina pectoris were recruited from two university hospitals in Western Norway from January 2000 to April 2004. Of these patients, 2573 (62.0%) were enrolled in the Western Norway B Vitamin Intervention Trial (WENBIT) which studied the prognostic impact of B-vitamin supplementation upon incident CV events and mortality (clinicaltrials.gov Identifier: NCT00354081) [11]. Patients for whom there was no BMI data (n = 3) were excluded from the study, as were underweight patients (BMI < 18.5 kg/m^2^) (n = 30).This left a total of 4131 subjects eligible for the analyses.

The study protocol met the mandate of the Helsinki Declaration, and was approved by the Western Norway Regional Committee for Medical and Health Research Ethics and the Norwegian Data Inspectorate. Written informed consent was obtained from all participants.

### Baseline data and biochemical analyses

Height, weight and blood pressure were measured at baseline by trained study personnel. BMI was calculated by dividing weight by height squared (kg/m^2^). Each patient provided information about medical history, risk factors and medications through a self-administered questionnaire, and all information was subsequently validated against medical records. Diabetes mellitus included type 1 and 2. Current smokers included those with self-reported current smoking, those who had quit smoking within <1 month and those with plasma cotinine >85 ng/mL [12]. Patients, who reported to have quit smoking > 1 month prior to inclusion and had plasma cotinine levels ≤85 ng/mL, were categorized as ex-smokers. Pulmonary disease included chronic obstructive lung disease, other chronic lung diseases and pulmonal hypertension. Cancer included active cancer with or without metastases. Family history of early coronary heart disease (CHD) encompassed those reporting to have at least one 1^st^ degree relative suffering from CHD before the age of 55 for men and 65 for women. Left ventricular ejection fraction (LVEF) was determined by ventriculography or echocardiography. The extent of CAD at angiography was scored as 0–3 as has previously been described [11]. Baseline coronary revascularisation procedures, after baseline angiography, included percutaneous coronary intervention (PCI) and coronary artery bypass graft surgery (CABG).

Blood samples were collected by study personnel prior to angiography and stored at -80˚C until analysis. Serum apolipoprotein A-1 (ApoA1), apolipoprotein B (ApoB) and lipoprotein (a) (Lp(a)) were analysed on the Hitachi 917 system (Roche Diagnostics, GmbH, Mannheim, Germany). Serum C-reactive protein (CRP) was measured using a latex, high sensitive assay (Behring Diagnostics, Marburg, Germany). Plasma cotinine was measured by liquid chromatography/tandem mass spectrometry [13]. Low density lipoprotein (LDL) cholesterol was calculated using the Friedewald formula [14] and estimated glomerular filtration rate (eGFR) was estimated using the Chronic Kidney Disease Epidemiology Collaboration formula [15].

### Follow-up and end points

The study participants were followed from angiography until they experienced one of the primary endpoints; AMI (fatal or non-fatal), death or till December 31^st^ 2006.

Information on clinical events was collected from The Western Norway Cardiovascular Registry and from the Cause of Death Registry at Statistics Norway as previously described [11]. An event was classified as fatal if death occurred within 28 days after onset. AMI was classified according to the diagnostic criteria of the revised AMI definition published in 2000 [16], and fatal strokes were classified according to diagnostic criteria published in 2001 [17]. Procedure-related non-fatal AMI occurring within 24 h of coronary angiography, PCI or CABG were not included in the end-point. CV death included causes of death coded I00-I99 or R96, according to the International Statistical Evaluation of Disease, Tenth Revision system. An endpoints committee adjudicated all events.

### Statistical analysis

Continuous variables are presented as means (standard deviation (SD)). Categorical variables are reported as counts (percentage). Non-normally distributed variables (diastolic blood pressure, serum creatinine, CRP, plasma glucose, serum triglycerides and Lp(a)) were log transformed. BMI groups were created using established BMI cut-offs; Normal weight (BMI 18.5-24.9 kg/m^2^), overweight (BMI 25–29.9 kg/m^2^) and obesity (BMI ≥30 kg/m^2^). Underweight patients (n = 30) were eliminated due to the possibility of reverse causation. Between group differences were tested by one-way analysis of variance (ANOVA) or independent samples t-test for continuous variables, and by chi square test for categorical variables. Post hoc tests were applied for multiple comparisons where appropriate.

The relationships between baseline BMI and subsequent risk of AMI, CV death and all-cause mortality were evaluated across BMI groups. Hazard ratios (HR) and 95% confidence intervals (CI) of endpoints associated with BMI categories were estimated with Cox proportional hazard models using the BMI normal weight category as reference. The time, in days, from angiography until endpoint (AMI, CV death and all-cause mortality) or end of study (December 31^st^ 2006) was used as time scale. Proportionality assumptions were tested by visual examination of log minus log plots and calculating Schoenfeld residuals. Covariates in the multivariate adjusted models were selected based on clinical relevance and the change-in-estimate method [18], with a limit for inclusion of 10% change in the risk ratio. The final multivariate model included gender, age (continuous), LVEF (%), current smoking (yes/no), angiotensin converting enzyme (ACE) -inhibitors (yes/no), loop diuretics (yes/no) and pulmonary disease (yes/no). Further adjustment of the multivariate Cox model did not alter the results in the total population or in gender stratified analyses; systolic and diastolic blood pressure (mmHg), diabetes mellitus (yes/no), previous AMI (yes/no), extent of significant CAD (0–3), serum creatinine levels (μmol/L), CRP (mg/L), total cholesterol (mmol/L), vitamin B6 (yes/no) or folate/B12 (yes/no) intervention status (data not shown).

Effect modifications by gender were investigated by including the product of gender and BMI categories as an interaction term in the multivariate adjusted Cox model.

All tests were 2-sided, and a p-value <0.05 was considered significant. Statistical analyses were performed with SPSS 17 (SPSS Inc, Chicago, IL) and R 2.14.2 (The R-Foundation for Statistical Computing, Vienna, Austria).

## Results

### Baseline characteristics

The cohort consisted of 4131 patients (72% males), and the mean (SD) age in the total population was 62 (10) years. Baseline characteristics across BMI groups are presented in Table 1. The mean (SD) BMI was 26.8 (3.9) kg/m^2^, and 34% of the patients had a BMI within the normal weight range, 48% were overweight and 19% were obese.

**Table 1:** **Baseline characteristics and laboratory findings according to BMI groups**^**a**^

	**Total n = 4131**	**Normal weight n = 1395**	**Overweight n = 1970**	**Obese n = 766**	**p-value**^**b**^
**Demographic characteristics**					
Male sex, n (%)	2989 (72.4)	959 (68.7)	1517 (77.0)	513 (67.0)	<0.001
Age (years)^c^	62 (10)	63 (11)	61 (10)	60 (10)	<0.001
**Clinical parameters**					
Systolic blood pressure (mmHg)	141 (20.7)	139 (21.0)	142 (20.6)	143 (20.3)	<0.001
Diastolic blood pressure (mmHg)	81.3 (10.4)	79.3 (10.3)	82.0 (10.1)	83.3 (10.7)	<0.001
Left ventricular ejection fraction (%)	64.0 (11.3)	63.7 (11.9)	64.4 (10.7)	63.6 (11.8)	0.08
**Cardiovascular risk factors, n (%)**					
Diabetes^d^	496 (12.0)	123 (8.8)	209 (10.6)	164 (21.4)	<0.001
Current smoker^e^	1061 (25.7)	417 (29.9)	469 (23.9)	175 (22.9)	<0.001
Ex smoker^f^	1933 (46.9)	571 (40.6)	975 (49.6)	387 (50.7)	<0.001
Family history of coronary heart disease^g^	1253 (31.1)	417 (30.8)	573 (29.7)	263 (35.1)	0.02
**Cardiovascular history, n (%)**					
Previous acute myocardial infarction	1670 (40.4)	564 (40.4)	784 (39.8)	322 (42.0)	0.56
Previous cerebrovascular disease	286 (6.9)	100 (7.2)	128 (6.5)	58 (7.6)	0.55
Previous peripheral vascular arterial disease	371 (9.0)	153 (11.0)	148 (7.5)	70 (9.1)	<0.01
Previous percutaneous coronary intervention	794 (19.2)	250 (17.9)	396 (20.1)	148 (19.3)	0.29
Previous coronary artery bypass graft surgery	477 (11.5)	150 (10.8)	238 (12.1)	89 (11.6)	0.49
**Extent of coronary artery disease at baseline as assessed by coronary angiography, n (%)**					
No significant coronary artery disease	1030 (24.9)	367 (26.3)	467 (23.7)	196 (25.6)	0.21
1-vessel disease	958 (23.2)	306 (21.9)	482 (24.5)	170 (22.2)	0.18
2-vessel disease	923 (22.3)	308 (22.1)	440 (22.3)	175 (22.8)	0.92
3-vesseldisease	1220 (29.5)	414 (29.7)	581 (29.5)	225 (29.4)	0.99
**Comorbidity at baseline, n (%)**					
Pulmonary disease	367 (8.9)	139 (10.0)	140 (7.1)	88 (11.5)	<0.001
Cancer	4 (0.1)	2 (0.1)	1 (0.1)	1 (0.1)	0.66
**Medication following baseline coronary angiography, n (%)**					
Acetylsalisylic acid	3376 (81.7)	1114 (79.9)	1638 (83.1)	624 (81.5)	0.05
Statins	3310 (80.1)	1064 (76.3)	1626 (82.5)	620 (80.9)	<0.001
β-blockers	2994 (72.5)	970 (69.5)	1456 (73.9)	568 (74.2)	0.01
ACE inhibitors	858 (20.8)	243 (17.4)	403 (20.5)	212 (27.7)	<0.001
Loop diuretics	447 (10.8)	127 (9.1)	182 (9.2)	138 (18.0)	<0.001
**Coronary revascularization following baseline coronary angiography, n (%)**					
Percutaneous coronary intervention	1348 (32.6)	432 (31.0)	663 (33.7)	253 (33.0)	0.26
Coronary artery bypass graft surgery	892 (21.6)	302 (21.6)	438 (22.2)	152 (19.8)	0.39
**Biochemical markers**					
Creatinine (μmol/L)	92.6 (31.1)	92.5 (33.4)	93.0 (32.0)	91.8 (23.4)	0.34
eGFR (mL/min)	87.8 (17.3)	86.2 (17.7)	88.5 (16.6)	88.9 (17.8)	<0.001
CRP (mg/L)	3.69 (7.17)	3.34 (7.93)	3.59 (6.67)	4.60 (6.85)	<0.001
Glucose (mmol/L)	6.35 (2.40)	5.93 (2.24)	6.32 (2.28)	7.20 (2.79)	<0.001
HbA1c (mmol/L)	6.22 (1.38)	6.12 (1.28)	6.18 (1.41)	6.51 (1.45)	<0.001
Hemoglobin (g/dL)	14.2 (1.24)	13.9 (1.24)	14.4 (1.18)	14.4 (1.29)	<0.001
ApoA1 (g/L)	1.32 (0.27)	1.37 (0.29)	1.30 (0.25)	1.27 (0.26)	<0.001
ApoB (g/L)	0.90 (0.25)	0.87 (0.24)	0.91 (0.24)	0.93 (0.26)	<0.001
Total cholesterol (mmol/L)	5.07 (1.17)	5.02 (1.14)	5.08 (1.18)	5.10 (1.19)	0.13
LDL cholesterol (mmol/L)	3.09 (1.03)	3.05 (1.02)	3.12 (1.00)	3.11 (1.10)	0.11
HDL cholesterol (mmol/L)	1.29 (0.38)	1.42 (0.43)	1.25 (0.34)	1.17 (0.32)	<0.001
Triglycerides (mmol/L)	1.78 (1.22)	1.43 (0.90)	1.86 (1.28)	2.22 (1.39)	<0.001
Lp(a) (mmol/L)	0.42 (0.39)	0.41 (0.39)	0.43 (0.39)	0.42 (0.39)	0.03
**WENBIT intervention trial, n (%)**	2560 (62.0)	797 (57.1)	1283 (65.1)	480 (62.7)	<0.001
B6, n (% of WENBIT participants)	1275 (49.8)	394 (49.4)	657 (51.2)	224 (46.7)	<0.01
Folate/B12, n (% of WENBIT participants)	1282 (50.1)	409 (51.3)	649 (50.6)	224 (46.7)	0.04

ACE, angiotensin converting enzyme; ApoA1, apolipoprotein A1; ApoB, apolipoprotein B; BMI, body mass index; CRP, c-reactive protein; eGFR, esitimated glomerular filtration rate; HDL, high density lipoprotein; LDL, low density lipoprotein; Lp(a), lipoprotein (a).

^a^Normal weight (BMI 18.5-24.9 kg/m^2^), overweight (BMI 25–29.9 kg/m^2^) and obese (BMI ≥ 30 kg/m^2^).

^b^Based on between group differences calculated by one-way analysis of variance (ANOVA) for continuous variables and Chi squared test for categorical variables.

^c^Mean (SD).

^d^Includes DM type 1 and 2.

^e^Smokers included self-reported current smoking, those who quit smoking within <1 month and patients with plasma cotinine >85 ng/mL.

^f^Patients reported to have quit smoking > 1 month prior to inclusion.

^g^Included those reporting to have at least one 1st degree relative suffering from CAD before the age of 55 for men and 65 for women.

Compared to the overweight and obese groups, the normal weight group was characterised by older age and a higher proportion of current smokers and subjects with a history of peripheral arterial disease. Mean blood pressure was lower in this group, as was the prevalence of diabetes. The extent of CAD at baseline did, however, not differ between the BMI categories. Compared to normal weight patients, overweight and obese patients were more often discharged with aspirin, statins and beta-blockers, while ACE inhibitors and loop diuretics were more often prescribed to obese patients.

The levels of eGFR, serum CRP, plasma glucose, HbA1c, Hb, serum ApoB, triglyceride and Lp(a) increased across incremental BMI groups, while serum ApoA1 and HDL cholesterol levels declined.

### Baseline characteristics according to gender

Among men, 32% were normal weight, 51% overweight and 17% obese, whereas the respective proportions were 38%, 40% and 22% among women. Mean (SD) BMI was 26.8 (3.7) kg/m^2^ among men and 26.8 (4.7) kg/m^2^ among women.

Baseline characteristics according to gender and BMI groups are presented in Table 2. Compared to women, men were generally younger, and there was an inverse relationship between age and BMI among men. Men had worse CV risk profile and more severe CAD, at baseline, than women. Correspondingly, men, compared to women, did more often undergo revascularisation procedures following baseline angiography and were more often discharged with medication.

**Table 2:** **Baseline characteristics and laboratory findings according to gender and BMI groups**^**a**^

	**Men**					**Women**					
	**Total**	**Normal weight**	**Overweight**	**Obese**		**Total**	**Normal weight**	**Overweight**	**Obese**		
	**n = 2989**	**n = 959**	**n = 1517**	**n = 513**	**p-value**^**b**^	**n = 1142**	**n = 436**	**n = 453**	**n = 253**	**p-value**^**c**^	**p-value**^**d**^
**Demographic characteristics**											
Age (years)^e^	61 (10)	63 (11)	61 (10)	59 (10)	<0.001	63 (10)	63 (19)	64 (11)	63 (10)	0.52	<0.001
**Clinical parameters**											
Systolic blood pressure (mmHg)	141 (20.4)	140 (20.5)	141 (20.3)	143 (20.4)	0.03	141 (21.6)	138 (22.1)	143 (21.6)	144 (20.2)	<0.01	0.94
Diastolic blood pressure (mmHg)	81.8 (10.4)	79.6 (10.1)	82.2 (10.0)	84.3 (11.1)	<0.001	80.0 (10.3)	78.4 (10.8)	80.9 (10.1)	81.0 (9.60)	<0.001	<0.001
Left ventricular ejection fraction (%)	63.1 (11.8)	62.6 (12.4)	63.7 (11.1)	62.1 (12.4)	<0.01	66.5 (9.81)	65.9 (10.3)	66.9 (9.21)	66.8 (9.93)	0.30	<0.001
**Cardiovascular risk factors, n (%)**											
Diabetes^f^	361 (12.1)	97 (10.1)	158 (10.4)	106 (20.7)	<0.001	135 (11.8)	26 (6.0)	51 (11.3)	58 (22.9)	<0.001	0.82
Current smoker^g^	803 (26.9)	305 (31.8)	375 (24.8)	123 (24.0)	<0.001	258 (22.7)	112 (25.7)	94 (20.8)	52 (22.7)	0.16	<0.01
Ex smoker^h^	1595 (53.4)	448 (46.7)	842 (55.6)	305 (59.6)	<0.001	338 (29.7)	123 (28.2)	133 (29.5)	82 (32.7)	0.47	<0.001
Family history of coronary heart disease^i^	844 (29.0)	258 (27.8)	417 (28.1)	169 (33.9)	0.03	409 (36.4)	159 (37.3)	156 (34.9)	94 (37.6)	0.69	<0.001
**Cardiovascular history, n (%)**											
Previous acute myocardial infarction	1346 (45.9)	429 (44.7)	668 (44.0)	249 (48.5)	0.20	324 (28.4)	135 (31.0)	116 (25.6)	73 (28.9)	0.21	<0.001
Previous cerebrovascular disease	203 (6.9)	69 (7.2)	101 (6.7)	33 (6.4)	0.82	83 (7.3)	31 (7.1)	27 (6.0)	25 (9.9)	0.16	0.59
Previous peripheral vascular arterial disease	276 (9.2)	114 (11.9)	115 (7.6)	47 (9.2)	<0.01	95 (8.3)	39 (8.9)	33 (7.3)	23 (9.1)	0.59	0.36
Previous percutaneous coronary intervention	641 (21.4)	193 (20.1)	340 (22.4)	108 (21.1)	0.39	153 (13.4)	57 (13.1)	56 (12.4)	40 (15.8)	0.42	<0.001
Previous coronary artery bypass graft surgery	394 (13.2)	116 (12.1)	205 (13.5)	73 (14.2)	0.44	83 (7.3)	34 (7.8)	33 (7.3)	16 (6.3)	0.77	<0.01
**Extent of coronary artery disease at baseline as assessed by coronary angiography, n (%)**											
No significant coronary artery disease	538 (18.0)	179 (17.7)	280 (18.5)	88 (17.2)	0.77	492 (43.1)	197 (45.2)	187 (41.3)	108 (42.7)	0.50	<0.001
1-vessel disease	704 (23.6)	218 (22.7)	373 (24.6)	113 (22.0)	0.38	254 (22.2)	88 (20.2)	109 (24.1)	57 (22.5)	0.38	0.37
2-vessel disease	731 (24.5)	236 (24.6)	360 (23.7)	135 (26.3)	0.50	192 (16.8)	72 (16.5)	80 (17.7)	40 (15.8)	0.80	<0.001
3-vesseldisease	1016 (34.0)	335 (34.9)	504 (33.2)	177 (34.5)	0.66	204 (17.9)	79 (18.1)	77 (17.0)	48 (19.0)	0.79	<0.001
**Comorbidity at baseline, n (%)**											
Pulmonary disease	276 (9.2)	96 (10.0)	115 (7.6)	65 (12.7)	0.04	91 (8.0)	43 (9.9)	25 (5.5)	23 (9.1)	<0.01	0.20
Cancer	4 (0.1)	2 (0.20)	1 (0.10)	1 (0.20)	0.59	0 (0.0)	0	0	0	-	0.22
**Medication following baseline coronary angiography, n (%)**											
Acetylsalisylic acid	2546 (85.2)	810 (84.5)	1306 (86.1)	430 (83.8)	0.13	830 (72.7)	304 (69.7)	332 (73.3)	194 (76.7)	0.34	<0.001
Statins	2492 (83.4)	759 (79.1)	1296 (85.4)	437 (85.2)	<0.001	818 (71.6)	305 (70.0)	330 (72.8)	183 (72.3)	0.61	<0.001
β-blockers	2229 (74.6)	684 (71.3)	1155 (76.1)	390 (76.0)	0.02	765 (67.0)	286 (65.6)	301 (66.4)	178 (70.4)	0.42	<0.001
ACE inhibitors	644 (21.5)	171 (17.8)	320 (21.1)	153 (29.8)	<0.001	214 (18.7)	72 (16.2)	83 (18.3)	59 (23.3)	0.08	0.05
Loop diuretics	297 (9.9)	90 (9.4)	128 (8.4)	79 (15.4)	<0.001	150 (13.1)	37 (8.5)	54 (11.9)	*59 (23.3)*	<0.001	<0.01
**Coronary revascularization following baseline coronary angiography, n (%)**											
Percutaneous coronary intervention	1065 (35.6)	327 (34.1)	545 (35.9)	193 (37.6)	0.38	283 (24.8)	105 (24.1)	118 (26.0)	60 (23.7)	0.72	<0.001
Coronary artery bypass graft surgery	731 (24.5)	248 (25.9)	363 (23.9)	120 (23.4)	0.46	161 (14.1)	54 (12.4)	75 (16.6)	32 (12.6)	0.15	<0.001
**Biochemical markers**											
Creatinine (μmol/L)	96.4 (31.7)	96.6 (30.5)	96.4 (34.9)	95.9 (23.3)	0.93	82.8 (26.5)	83.9 (36.7)	81.4 (14.5)	83.4 (21.5)	0.35	<0.001
eGFR (mL/min)	89.3 (17.0)	87.5 (16.6)	89.7 (16.6)	91.2 (16.9)	<0.001	84.1 (17.3)	83.4 (17.6)	84.6 (16.1)	84.2 (18.7)	0.58	<0.001
CRP (mg/L)	3.66 (7.59)	3.55 (8.91)	3.58 (6.99)	4.07 (6.49)	0.39	3.79 (5.93)	2.88(5.17	3.62 (5.43)	5.66 (7.42)	<0.001	0.59
Glucose (mmol/L)	6.41 (2.38)	6.08 (2.42)	6.33 (2.14)	7.26 (2.77)	<0.001	6.20 (2.46)	5.60 (1.75)	6.30 (2.68)	7.06 (2.82)	<0.001	0.01
HbA1c (mmol/L)	6.17 (1.39)	6.16 (1.11)	6.40 (1.52)	6.62 (1.49)	<0.001	6.36 (1.38)	6.10 (1.36)	6.11 (1.37)	6.46 (1.43)	<0.001	<0.001
Hemoglobin (g/dL)	14.6 (1.16)	14.2 (1.21)	14.7 (1.07)	14.8 (1.16)	<0.001	13.4 (1.05)	13.3 (1.04)	13.5 (1.04)	13.5 (1.05)	<0.01	<0.001
ApoA1 (g/L)	1.26 (0.25)	1.31 (0.27)	1.25 (0.23)	1.20 (0.23)	<0.001	1.46 (0.27)	1.49 (0.28)	1.45 (0.26	1.42 (0.27)	<0.01	<0.001
ApoB (g/L)	0.90 (0.25)	0.86 (0.24)	0.90 (0.24)	0.93 (0.27)	<0.001	0.92 (0.25)	0.88 (0.24)	0.94 (0.26)	0.94 (0.25)	<0.01	<0.01
Total cholesterol (mmol/L)	4.98 (1.17)	5.18 (1.12)	5.39 (1.14)	5.25 (1.16)	0.30	5.28 (1.14)	5.18 (1.12)	5.39 (1.14)	5.25 (1.16)	0.02	<0.001
LDL cholesterol (mmol/L)	3.05 (1.02)	3.03 (1.01)	3.06 (0.98)	3.07 (1.13)	0.64	3.19 (1.03)	3.08 (1.02)	3.30 (1.03)	3.18 (1.04)	<0.01	<0.001
HDL cholesterol (mmol/L)	1.22 (0.33)	1.33 (0.38)	1.19 (0.29)	1.09 (0.26)	<0.001	1.47 (0.42)	1.57 (0.44)	1.45 (0.39)	1.34 (0.37)	<0.001	<0.001
Triglycerides (mmol/L)	1.85 (1.28)	1.48 (0.97)	1.91 (1.30)	2.32 (1.52)	<0.001	1.62 (1.03)	1.34 (0.72)	1.67 (1.18)	2.00 (1.07)	<0.001	<0.001
Lp(a) (mmol/L)	0.41 (0.37)	0.43 (0.41)	0.48 (0.45)	0.47 (0.40)	0.79	0.46 (0.42)	0.43 (0.41)	0.48 (0.45)	0.47 (0.40)	0.11	<0.01
**WENBIT intervention trial, n (%)**	2047 (68.5)	614 (64.0)	1072 (70.7)	361 (70.4)	<0.01	513 (44.9)	183 (42.0)	211 (46.6)	119 (47.0)	0.29	<0.001
B6, n (% of WENBIT participants)	1043 (34.9)	320 (52.1)	553 (51.9)	170 (47.1)	0.19	239 (20.9)	89 (48.6)	96 (45.5)	54 (45.4)	0.94	<0.001
Folate/B12, n (% of WENBIT participants)	1031 (34.5)	301 (49.0)	555 (51.8)	175 (48.5)	0.03	244 (21.4)	93 (50.8)	102 (48.3)	49 (41.2)	0.92	<0.001

ACE, angiotensin converting enzyme; ApoA1, apolipoprotein A1; ApoB, apolipoprotein B; BMI, body mass index; CRP, c-reactive protein; eGFR, esitimated glomerular filtration rate; HDL, high density lipoprotein; LDL, low density lipoprotein; Lp(a), lipoprotein (a).

^a^Normal weight (BMI 18.5-24.9 kg/m^2^), overweight (BMI 25–29.9 kg/m^2^) and obese (BMI ≥ 30 kg/m^2^).

^b^Based on differences between BMI groups among men, and was calculated by ANOVA for continuous variables and Chi squared test for categorical variables for differences between men and women.

^c^Based on differences between BMI groups among women, and was calculated by ANOVA for continuous variables and Chi squared test for categorical variables for differences between men and women.

^d^Based on between group differences in men vs. women, and was calculated by independent samples t-test for continuous variables and Chi squared test for categorical variables.

^e^Mean (SD).

^f^Includes DM type 1 and 2.

^g^Smokers included self-reported current smoking, those who quit smoking within <1 month and patients with plasma cotinine >85 ng/mL.

^h^Patients reported to have quit smoking > 1 month prior to inclusion.

^i^Included those reporting to have at least one 1st degree relative suffering from CAD before the age of 55 for men and 65 for women.

### Follow-up and end-points

During the follow-up period (mean (SD) 4.8 (1.4) years), 337 (8.2%) patients experienced an AMI, of which 101 (30%) were fatal. A total of 302 (7.3%) patients died, of whom 165 (55%) died from cardiovascular causes.

There were statistically significant multivariate adjusted interactions between gender and BMI categories with regards to risk of incident AMI (p-int = 0.011) and CV-death (p-int = 0.031), but not to all-cause mortality (p-int = 0.427).

A total of 115 (8.2%) normal weight patients, 127 (6.4%) overweight patients and 60 (7.8%) obese patients died. The risk of all-cause mortality did not differ significantly between BMI categories in any analyses; compared to the normal weight group, the multivariate adjusted HR (95% CI) was 0.95 (0.74, 1.23) in the overweight group and 1.16 (0.84, 1.60) in the obese group.

Analyses were repeated in subgroups of patients with significant CAD or without diabetes only, and the results were not significantly different from those reported (data not shown).

### Gender stratified analyses

A total of 267 (8.9%) men and 70 (6.1%) women suffered an AMI. Further, 241 (8.1%) men and 61 (5.3%) women died, whereof 132 (55%) male deaths and 33 (54%) female deaths were characterised as CV deaths.

Obese men had a significantly higher multivariate adjusted risk of both incident AMI; HR 1.80 (1.28, 2.52), and CV death; HR 1.60 (1.00, 2.55), compared to normal weight men (Table 3).

**Table 3:** **BMI groups**^**a **^**and risk of acute myocardial infarction and cardiovascular death in men**

**Model**	**Acute myocardial infarction**				**Cardiovascular death**			
	**Events, n (%)**	**HR**	**95% CI**	**p-value**	**Events, n (%)**	**HR**	**95% CI**	**p-value**
**Univariate**								
Normal weight	84 (8.8)	1.00			51 (5.3)	1.00		
Overweight	117 (7.7)	0.88	0.66, 1.16	0.37	49 (3.2)	0.61	0.41, 0.90	0.01
Obese	66 (12.9)	1.51	1.09, 2.08	0.01	32 (6.2)	1.19	0.76, 1.85	0.45
**Multivariate adjusted**^**b**^								
Normal weight		1.00				1.00		
Overweight		1.11	0.84, 1.48	0.47		0.85	0.57, 1.28	0.44
Obese		1.80	1.28, 2.52	<0.01		1.60	1.00, 2.55	0.05

BMI, body mass index; CI, confidence interval; HR, hazard ratio.

^a^Normal weight (BMI 18.5-24.9 kg/m^2^), overweight (BMI 25–29.9 kg/m^2^) and obese (BMI ≥ 30 kg/m^2^).

^b^Age (continuous), current smoking (yes/no), left ventricular ejection fraction (%), pulmonary disease (yes/no), angiotensin converting enzyme-inhibitors (yes/no) and loop diuretics (yes/no).

Overweight women had a significantly lower multivariate adjusted risk of AMI; HR 0.56 (0.33, 0.98), compared to normal weight women (Table 4). By contrast, the multivariate adjusted HR for AMI between normal weight women and obese women did not differ significantly.

**Table 4:** **BMI groups**^**a **^**and risk of acute myocardial infarction and cardiovascular death in women**

**Model**	**Acute myocardial infarction**				**Cardiovascular death**			
	**Events, n (%)**	**HR**	**95% CI**	**p-value**	**Events, n (%)**	**HR**	**95% CI**	**p-value**
**Univariate**								
Normal weight	33 (7.6)	1.00			16 (3.7)	1.00		
Overweight	21 (4.6)	0.61	0.35, 1.05	0.08	13 (2.9)	0.78	0.38, 1.63	0.51
Obese	16 (6.3)	0.84	0.46, 1.53	0.57	4 (1.6)	0.43	0.14, 1.28	0.13
**Multivariate adjusted**^**b**^								
Normal weight		1.00				1.00		
Overweight		0.56	0.33, 0.98	0.04		0.71	0.34, 1.50	0.37
Obese		0.80	0.43, 1.47	0.46		0.38	0.21, 1.16	0.09

BMI, body mass index; CI, confidence interval; HR, hazard ratio.

^a^ Normal weight (BMI 18.5-24.9 kg/m^2^), overweight (BMI 25–29.9 kg/m^2^) and obese (BMI ≥ 30 kg/m^2^).

^b^ Age (continuous), current smoking (yes/no), left ventricular ejection fraction (%), pulmonary disease (yes/no), angiotensin converting enzyme-inhibitors (yes/no) and loop diuretics (yes/no).

## Discussion

### Principal findings

In this large longitudinal prospective cohort study of more than 4000 patients with suspected stable angina pectoris, we demonstrate that obese male patients had a 1.8 fold and 1.6 fold increased risk of incident AMI and CV death compared to normal weight men. By contrast, compared to normal weight women, obese women had similar risk of AMI and CV death, while overweight women had nearly half the risk of incident AMI. The risk of all-cause mortality associated with BMI was similar among men and women, and did not differ significantly across BMI categories.

### BMI and risk of AMI, CV death and all-cause mortality in men and women

Strong associations between overweight/obesity and risk of CVD and death have been demonstrated in the general population [19,20]. By contrast, several studies of patients with CAD have demonstrated that overweight and/or obese patients may have a better morbidity and mortality prognosis than their leaner counterparts; although as one recent review points out, this observation is not supported by all [3].

Only a few studies of patients with CAD have examined the association between BMI and risk of CV events and mortality in men and women separately. Our finding of an increased risk of cardiovascular events among obese men are in accordance with the results from a previous US study of patients with stable CVD, whereof 85% had CHD, as well as with a multi-ethnic sample study of patients with established CAD [5,6]. However, while these studies did not observe any significant associations between BMI and risk of major adverse coronary events in women, we report a nearly halved adjusted risk of AMI among overweight women as compared to their normal weight counterparts.

In the present study, there was no interaction between BMI and gender with regards to all-cause mortality. Moreover, the risk of death did not differ between BMI groups. These findings are in accordance with a previous study conducted among European patients with CAD [21]. To the best of our knowledge only two studies, of patients with CAD, have examined the association between BMI and all-cause mortality in men and women separately [7,8]. First, a study of Danish patients with AMI showed that normal weight, overweight and obese men had similar risk of death, whereas overweight women had a slightly decreased risk (HR (95% CI); 0.78 (0.68, 0.90)) of death, as compared to their normal weight counterparts. Furthermore, in a follow up study of the CADILLAC trial, they observed significantly lower in-hospital mortality (0.9% vs. 2.7%), 30 days (1.1% vs. 3.8%) and 1- year (1.8% vs. 7.5%) mortality in obese patients with AMI undergoing PCI when compared to normal weight patients. Statistical significance was, however, only reached in males.

### Possible explanations

BMI is often used to quantify overweight and obesity owing to a high fat percentage correlation, but does not account for fat distribution. Men have a tendency to store excessive fat in visceral fat deposits, whereas women usually store fat in peripheral subcutaneous distributions [4]. Excessive visceral fat is associated with an increased risk of developing metabolic syndrome, putting men at a greater risk of developing CVD, while subcutaneous fat in the femoral-gluteal region may be associated with a more favourable CV risk profile [22]. Furthermore, overweight and obese postmenopausal women may benefit from the increase in circulating levels of estrogen produced by the adipose tissue [23,24].

Moreover, at baseline, men were more often affected by CV risk factors and had more severe CAD. Inclusion of these potential confounding variables in stratified multivariate analyses did not alter our results. Differing health status at baseline is thus unlikely to be the cause of the observed gender interaction.

### Strengths and limitations of the study

The main strength of the present study is its, well defined population with complete follow up of clinical endpoints. Limitations include the single baseline measurements of BMI and other time dependent cofactors such as medication. We did not have sufficient data on possible confounders such as physical activity, socioeconomic status or cardio- respiratory fitness and intentional vs. unintentional weight loss, and thus we cannot exclude the possibility that residual confounding from unmeasured causal factors unevenly distributed between BMI groups may have influenced our results. Unfortunately, we did not have data on recent weight loss prior to inclusion, but as underweight patients (BMI <18.5 kg/m^2^) were excluded, and adjustment for possible confounders such as cancer, pulmonary disease, extent of significant CAD and LVEF did not significantly alter our results, reverse causation is unlikely. BMI was positively associated with common obesity related characteristics such as higher blood pressure, diabetes, an unfavourable lipid profile, higher eGFR and CRP. Adjustment for these variables did not have a significant effect on our results, but we would in any case not include these variables in a final multivariate adjusted survival model because of the possibility of over-adjustment bias. We did, however, adjust for use of ACE inhibitors and loop diuretics as a proxy of heart failure, and there is the possibility that these variables may have mediated some of the effect of BMI.

It has previously been suggested that BMI is an inadequate marker of overweight and obesity in patients with CAD [25], with waist circumference or waist to hip ratio suggested as better predictors of cardiovascular events, especially in women [6,26]. Studies supporting an obesity paradox have almost exclusively used BMI as an index of obesity [3]. We thus suspect that the diverging findings among such studies may be the result of BMI’s inadequacy as a quantifier of true body fatness and fat distribution.

Given that there were relatively few females in the study population and the event rate was low, we thus had a low statistical power to by which to detect the possible effects of BMI on risk of events among women. Further, we cannot rule out that the relatively lower incidence rate of AMI among women is a result of detection bias; women, compared to men, are more likely to experience atypical symptoms of AMI and may consequently delay seeking medical care for symptoms or be misdiagnosed by healthcare providers [27]. Finally, the inclusion of predominantly white subjects limits the ability to generalise our findings to non-white populations.

## Conclusion

Among 4131 men and women with suspected stable angina pectoris, obese men carried an 80% and 60% higher risk of AMI and CV death, respectively, compared to normal weight men, whereas being overweight, compared to normal weight, was associated with a 50% lower risk of AMI among women. These findings may potentially explain some of the result variation in studies reporting on the obesity paradox, with further investigation of the interaction between gender and BMI in terms of risk of CV events and mortality therefore warranted.

## Competing interests

The authors declare that they have no competing interests.

## Authors’ contributions

ON conceived of the study and contributed to the study design; GFTS, EKRP, HSH and ON conducted research; HB, RS analyzed data or performed statistical analysis; HB, JKH, GFTS, JH and ON wrote the paper; HB had primary responsibility for final content; HB, JKH, JH and ON interpreted data; HB, JKH, GFTS, EKRP, HSH, JH and ON critically revised the manuscript. All listed authors take responsibility for all aspects of the reliability and freedom from bias of the data presented and their discussed interpretation. All authors read and approved the final manuscript.

## Pre-publication history

The pre-publication history for this paper can be accessed here:

http://www.biomedcentral.com/1471-2261/14/68/prepub
